# Hexokinase inhibition using D-Mannoheptulose enhances oncolytic newcastle disease virus-mediated killing of breast cancer cells

**DOI:** 10.1186/s12935-020-01514-2

**Published:** 2020-08-28

**Authors:** Ahmed Ghdhban Al-Ziaydi, Ahmed Majeed Al-Shammari, Mohammed I. Hamzah, Haider Sabah kadhim, Majid Sakhi Jabir

**Affiliations:** 1 Department of Medical Chemistry, College of Medicine, University of Al-Qadisiyah, Al Diwaniyah, Iraq; 2 Experimental Therapy, Iraqi Center for Cancer and Medical Genetics Research, Mustansiriyah University, Baghdad, Iraq; 3 College of Medicine, University of Al-Nahrain, Baghdad, Iraq; 4 Department of Microbiology, College of Medicine, Al-Nahrain University, Baghdad, Iraq; 5 Division of Biotechnology, Department of Applied Science, University of Technology, Baghdad, Iraq

**Keywords:** Cytotoxicity, Anticancer therapy, Warburg effect, Hexokinase inhibitor, Pyruvate

## Abstract

**Background:**

Most cancer cells exhibit increased glycolysis and use this metabolic pathway cell growth and proliferation. Targeting cancer cells’ metabolism is a promising strategy in inhibiting cancer cell progression. We used D-Mannoheptulose, a specific hexokinase inhibitor, to inhibit glycolysis to enhance the Newcastle disease virus anti-tumor effect.

**Methods:**

Human breast cancer cells were treated by NDV and/or hexokinase inhibitor. The study included cell viability, apoptosis, and study levels of hexokinase enzyme, pyruvate, ATP, and acidity. The combination index was measured to determine the synergism of NDV and hexokinase inhibitor.

**Results:**

The results showed synergistic cytotoxicity against breast cancer cells by combination therapy but no cytotoxic effect against normal cells. The effect was accompanied by apoptotic cell death and hexokinase downregulation and inhibition to glycolysis products, pyruvate, ATP, and acidity.

**Conclusions:**

The combination treatment showed safe significant tumor cell proliferation inhibition compared to monotherapies suggesting a novel strategy for anti-breast cancer therapy through glycolysis inhibition by hexokinase downregulation.

## Background

Cancer cell generally relies on aerobic glycolysis due to hypoxia, mitochondrial dysfunction, and the malignant transformation that lead to glycolysis pathway-dependent for ATP generation [1]. This phenomenon is described as the Warburg effect where cancer cells require high amounts of glucose to support the metabolic function and produce energy [2]. Cancer cells mainly produce energy by increasing the rate of glycolysis by 200 times higher than that in the normal cells of origin; this increment is succeeded by lactate fermentation in the cytosol of the cell regardless of the abundant oxygen supply [3]. The uptake of glucose in normal tissue is less than that in cancer cells. This difference can be used as a target for cancer therapy [4]. Breast-cancer stem cells are fermentative glycolysis dependent, and that makes it sensitive to glycolysis inhibitors [5].

The first and rate-limiting step of glycolysis controlled by hexokinase (HK) enzyme is glucose phosphorylation to glucose-6-phosphate (Glu-6-P), which is later employed to generate two ATP molecules [6]. In mammalian tissues, there are four major hexokinases are expressed, designated as HK1, HK2, HK3, and HK4 [7]. HK1 and HK2 are the main hexokinases that are important in cell survival. Mammalian adult tissues mostly expressed HK1 isoform, while HK2 is expressed abundantly only in dew adult tissues such as cardiac muscles, skeletal, and adipose [6]. It is found that HK2 is expressed in many types of cancers to promotes its growth through an increased glycolytic flux [8]. Brown et al. [9] found that breast cancers were HK2-positive in 79% of studied tumors. HKII status in breast cancer tissue sections was significantly related to poor prognosis and relapse of breast cancers [10]. Moreover, tamoxifen-resistant breast cancer cells MCF-7 showed upregulation of HK2 and mTOR that was accompanied by an enhanced glycolysis process. These findings make HK2 as a possible target to be blocked to overcome resistance to tamoxifen [11]. And for paclitaxel resistance [12]. Furthermore, HK2 overexpression in ovarian cancer cells induces cisplatin resistance [13]. Therefore, targeting hexokinase-2 will block glucose metabolism in cancer cells, which may inhibit its proliferation with minimum side effects reported [14]. The non-metabolizable glucose analog D-Mannoheptulose (MH) inhibits hexokinase, the first enzyme in glycolysis, with anticancer effect [15, 16], which lead to block cellular energy production [17]. D-Mannoheptulose (MH) accumulates in avocado leaves and occasionally in avocado fruit [18]. Mannoheptulose described by Dakubo [19] as an inhibitor for HK II with many other agents including lonidamine, 3-BrPA, 2-deoxyglucose, and 5-thioglucose.

On the other hand, cancer is still a very difficult disease to treat. Therefore, we need to find an effective and selective treatment approach that can destroy cancer cells, such as oncolytic virotherapy [20]. One of the first oncolytic Viruses discovered since the late 20th century is the Newcastle disease virus (NDV) [21]. The AMHA1 NDV is an avirulent attenuated strain of avian paramyxovirus, which is enveloped, non-segmented, negative-sense RNA viruses [22]. AMHA1 possesses onco-tropism characteristics [23]. NDV induced apoptosis via caspase-dependent and caspase-independent pathways [24, 25]. Deng et al. [26] found in a proteomic study that NDV downregulate phosphoglycerate kinase (PGK) expression in the NDV-infected chicken peripheral blood mononuclear cells (PBMCs), PGK is a glycolytic enzyme that participates in the glycolytic pathway, which suggests that NDV may restrict the infected cell glycolytic pathway. Its reported that interfering with cancer cells metabolism through glycolysis inhibition may enhance oncolytic virotherapy activity [27]. A recent study showed that using 2-deoxyglucose (2DG) to block glycolysis or through restricting glucose amount will enhance oncolytic adenoviruses activity in permissive and poorly permissive cancer cells [28]. Furthermore, pyruvate dehydrogenase kinase inhibition showed to improve reovirus oncolytic anti-tumor efficacy in several cancer types [29]. We reported previously that 2DG would enhance oncolytic NDV against breast cancer cells through glycolysis inhibition by downregulation of glyceraldehyde3-phosphate (GAPDH) [30]. Here, we investigate using D-Mannoheptulose, a hexokinase inhibitor to increase breast cancer cells sensitivity to oncolytic NDV, and the proposed mechanisms of this combination using glycolysis products analysis in breast cancer cells NDV-mediated oncolysis correlates with the disturbance within the metabolism.

## Materials and methods

### NDV propagation

NDV (Iraqi AMHA1 strain) was provided by the Experimental Therapy Department/Iraqi Center of Cancer and Medical Genetics Research (ICCMGR), Mustansiriyah University, Baghdad, Iraq. A stock of attenuated NDV was propagated in embryonated chicken eggs (Al-Kindi Company, Baghdad, Iraq), harvested from allantoic fluid, and then purified from debris through centrifugation (3000 rpm, 30 min at 4 °C). NDV was quantified through a hemagglutination test, aliquoted, and stored at − 80 °C. Viral titers were determined based on 50% tissue culture infective dose titration on Vero cells following standard procedure [31].

### Cell lines and cell culture

The Cell Bank Unit provided the estrogen, progesterone receptors negative AMJ13 human breast cancer cell line [32], The estrogen, progesterone receptors positive MCF-7 human breast cancer cell line, and normal rat embryo fibroblast cell line (REF), Experimental Therapy Department, ICCMGR, Baghdad, Iraq. AMJ13 and REF cell lines were cultured in RPMI-1640 medium. In contrast, MCF-7 cell was cultured in MEM (US Biological, USA) supplemented with 10% (v/v) fetal bovine serum (FBS) (Capricorn-Scientific, Germany) and 1% (v/v) penicillin–streptomycin (Capricorn-Scientific, Germany) and incubated in a humidified atmosphere of 5% CO_2_ at 37 °C. Exponentially growing cells were used for experiments.

### Cytotoxicity assay

Cells were seeded at a density of 1 × 10^4^ cells/well in a 96-well microplate and incubated at 37 °C for 72 h until monolayer confluence was achieved as observed under an inverted microscope. Cytotoxicity was investigated by using 3-(4, 5-dimethylthiazol-2-yl)-2, 5-diphenyltetrazolium bromide (MTT) assay. The cells were exposed to a range of diluted concentrations of MH (13.125, 26.25, 52.5, 105, 210, 840, and 1680 μg/ml) (Santacruz Biotechnology, USA) and NDV over a range of diluted multiplicities of infection (MOI, 0.1, 0.2, 0.4, 0.8, 1.6, 3.2, 6.4, and 12.8) for IC50 determination. After 72 h, each well received 50 μl of MTT dye solution (2 mg/ml), incubated for 3 h, and solubilized with 100 μl of dimethyl sulfoxide (DMSO). The plates were incubated for 15 min. The optical density values of treated and untreated cells were measured at 492 nm with an ELISA plate reader [32]. Exactly 200 µL of cell suspension was seeded in 96-well microtitration plates at the density of 1 × 10^4^ cells/ml and incubated for 72 h at 37 °C to visualize cell morphology under an inverted microscope. The medium was removed, and NDV and MH were added. The plates were stained with 50 µl of crystal violet and incubated at 37 °C for 15 min. Finally, the stain was removed through gentle washing with tap water. The cells were observed under an inverted microscope at 40 × magnification and photographed by using a digital camera. The endpoint parameter for each cell line included the inhibition rate of cell growth (cytotoxicity  %), which was calculated as follows:

$${\text{Cytotoxicity }}\% = \, \left( {{\text{OD}}_{\text{Control}} {-}{\text{ OD}}_{\text{sample}} } \right) \, /{\text{ OD}}_{\text{Control}} \times 100,$$

where OD _control_ is the mean optical density of untreated wells, and OD _Sample_ is the optical density of treated wells [23].

### Combined cytotoxicity assays and Chou–Talalay analysis

The doses for this experiment were selected from the previous cytotoxicity assay results of IC50 determination. We took concentrations around the IC50 value for NDV and MH in cancer cells. The AMJ13, MCF7, and REF cell lines were seeded at the density of 1 × 10^4^ cells/well into 96-well plates and incubated overnight. NDV was added first at MOI of 0.3, 1, and 2, followed by MH at the indicated concentrations (62.5, 125, and 250 μg/ml) through serial dilution for the growth inhibition test. Growth inhibition was measured after 72 h of infection through MTT assay, as described earlier. The assay was performed in triplicate. NDV and MH were studied at nonconstant ratios to determine synergism. The Chou–Talalay combination index (CI) was calculated using CompuSyn software (CombuSyn Inc., Paramus, NJ, USA) to analyze the combination of NDV and MH. The unfixed ratios of NDV and MH and mutually exclusive equations were used to determine the combination index (CI). CI values of 0.9–1.1, < 0.9, and > 1.1 indicate additive, synergism, and antagonism, respectively [33].

### Assessment of apoptosis (propidium iodide/acridine orange assay)

Propidium iodide/acridine orange (PI/AO) dual staining was used to measure the apoptotic rates in infected and control breast cancer cells. The cells were seeded at the density of 7000 cells/well in a 96-well plate the night before treatment and then treated with MH of 62.5 µg/ml and NDV MOI of 2 in a 37 °C incubator for 72 h before PI/AO staining. Then, 1 ml of cell suspension was used for conventional PI/AO staining (10 μl AO + 10 μl PI + 1 ml PBS). Exactly 50 μl of stain mixture (AO/PI) was added to the tested wells, which were allowed to stand for 30 s at room temperature (RT). The dye was then discarded. Photographs were taken directly under a Leica fluorescent microscope [34].

### Cell treatment for glycolysis pathway evaluation

The AMJ13, MCF7, and REF cell lines were seeded in 96-well cell culture plates at a density of 1 × 10^4^ cells/ml and incubated overnight. After confluence, the cells were exposed to 62.5 µg/ml MH and NDV at MOI 2 alone or in combination and compared with the control (untreated cell) [35] after 72 h cell lysate were collected, normalized, and stored at − 86 °C until used.

### HK activity assay

HK activity levels were measured in treated and untreated cell samples through a colorimetric method by using a hexokinase Assay Kit (ElabScience, USA) per the manufacturer’s recommendation. The kit detection principle, glucose is converted to glucose-6-phosphate by hexokinase; the glucose-6-phosphate is oxidized by glucose-6-phosphate dehydrogenase (G6PD) to produce NADH, which reduces the colorless substrate to the colored solution, HK activity can be calculated by determining the absorbance at 340 nm.

### Pyruvate assay

Pyruvate levels were measured using a colorimetric assay kit according to the manufacturer’s instructions (ElabScience, USA). The detection principle is that pyruvic acid reacts with chromogenic reagent to form a reddish-brown solution. The color intensity is directly related to the pyruvate content. The OD values were measured of each sample at 505 nm using a spectrophotometer.

### ATP assay

ATP was measured through a colorimetric method by using an ATP assay kit (ElabScience, USA). Cell culture samples were prepared as follows: treated and untreated cell samples were collected and centrifuged at 1000–1500 r/min for 10 min. The supernatant was removed, and the cell sediment was retained (approximately 10^6^/ml cells). The cell suspension was prepared with 0.3–0.5 ml of boiled double-distilled water, allowed to stand in a boiling water bath for 10 min, blended, and finally extracted for 1 min. The sample was centrifuged at 3500 r/min for 10 min, and the supernatant was obtained for detection. The OD values of each tube were measured at 636 nm, following the manufacturer’s recommendation.

### PH measurements

The cancer cells were seeded at a density of 1 × 10^4^ cells/plate in a 96-well microplate and incubated at 37 °C for 72 h until monolayer confluence was achieved as observed under an inverted microscope. The cells were exposed to MH (62.5 μg/ml) and NDV (MOI 2) and incubated at 37 °C for 72 h. The media supernatant was collected. The pH values were measured by using PH meter and litmus papers and compared with those of the control [36].

### Statistical analysis

All results were presented as SD and mean ± SEM. unpaired *t* test and statistical analysis were performed with statistical software Excel version 10, GraphPad Prism version 7 (USA). CompuSyn software was used to compare the difference between groups under different conditions. The level of significance was set at P < 0.05.

## Results

### Cytotoxicity of NDV and MH against breast cancer and normal cell lines

**Fig. 1:**
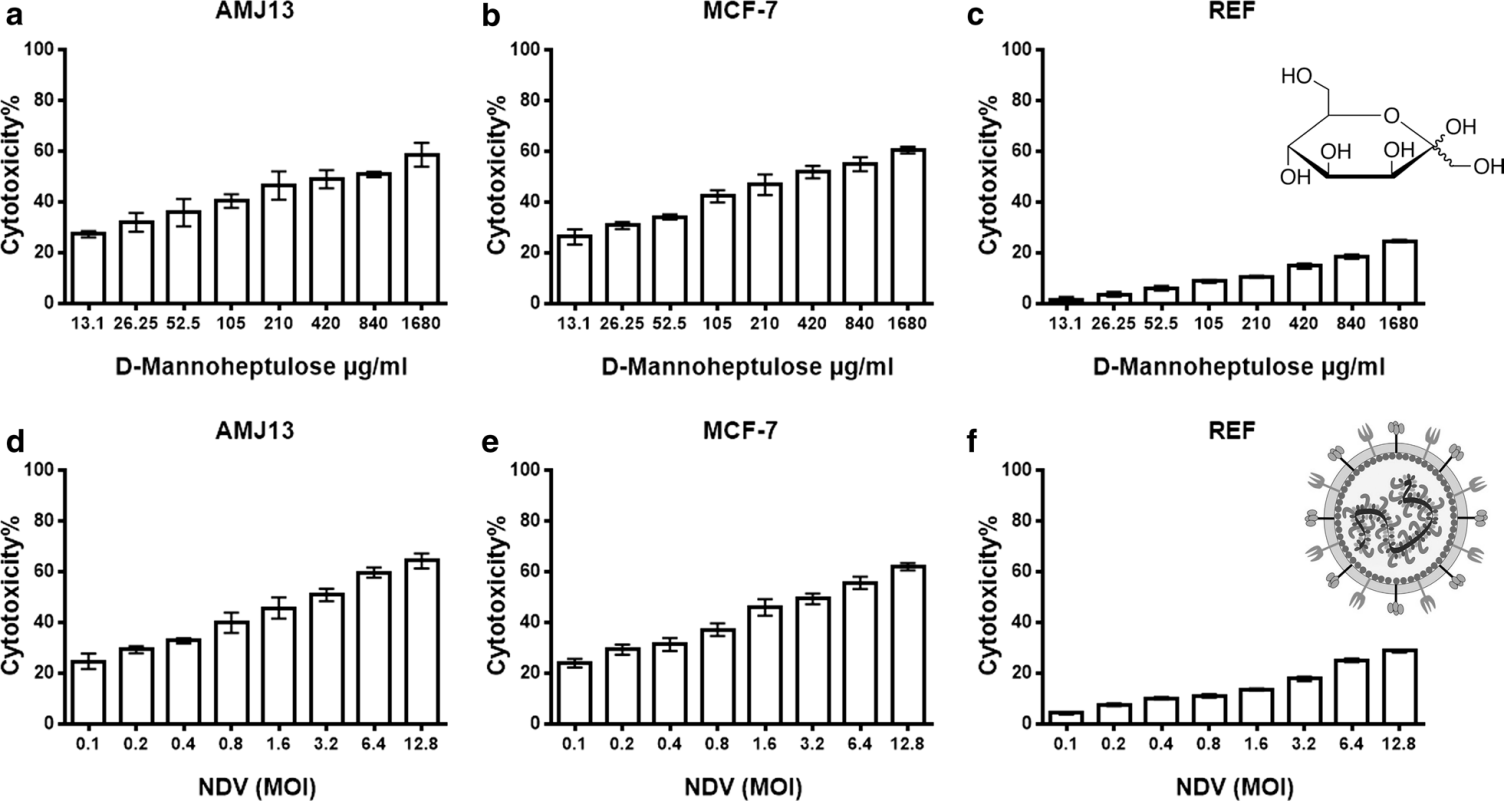
MH and oncolytic AMHA1 NDV are cytotoxic against human AMJ13 and MCF-7 breast cancer cells, but not cytotoxic to normal embryonic REF cells. The cells were treated with (**a**–**c**) D-Mannoheptulose (MH) (13.125, 26.25, 52.5, 105, 210, 840, and 1680 μg/ml) or (**d**–**f**) NDV (MOI 0.1, 0.2, 0.4, 0.8, 1.6, 3.2, 6.4, and 12.8) for 72 h. cytotoxicity was investigated using MTT assay and showed that IC50 values of MH (486.9 µg REF, 124.7 µg AMJ13, and 122.6 µg MCF7) and IC50 values for NDV MOI (57.5 REF, 1.648 AMJ13 and 1.561 MCF-7). All data shown are mean ± SEM from three independent experiments

MTT cytotoxicity assay was used to evaluate the effect against cancer and normal cells of different concentrations of MH and over a range of MOI of NDV (Fig. 1). There were no noticeable percentages of cytotoxicity (CT%) for MH against normal REF cells, as the CT% ranged from 1.67 to 24.72% at higher concentrations. While there was higher cytotoxicity against breast cancer cells ranged from 27.29 to 58.64% for AMJ13 cells; and 26.26% to 60.49% for MCF-7 after MH treatment (Fig. 1a–c). NDV virotherapy did not induce a cytotoxic effect against normal embryonic REF cells (Fig. 1f). Breast cancer cells were more sensitive to NDV virotherapy as the CT% ranged from 24.69% to 64.26% for AMJ13; and 23.95% to 62.02% for MCF-7 cell line after NDV treatment (Fig. 1d–f). The cytotoxicity assay analysis showed that IC50 values of MH (486.9 µg REF, 124.7 µg AMJ13, and 122.6 µg MCF7) and IC50 values for NDV MOI (57.5 REF, 1.648 AMJ13, and 1.561 MCF-7). Therefore, we chose IC50 related doses of the MH and NDV for the combination study, (0.3, 1, 2 MOI) for NDV and (62.5, 125, and 250 μg/ml) for MH.

### Combination cytotoxicity assays and Chou–Talalay analysis of cell lines

**Fig. 2:**
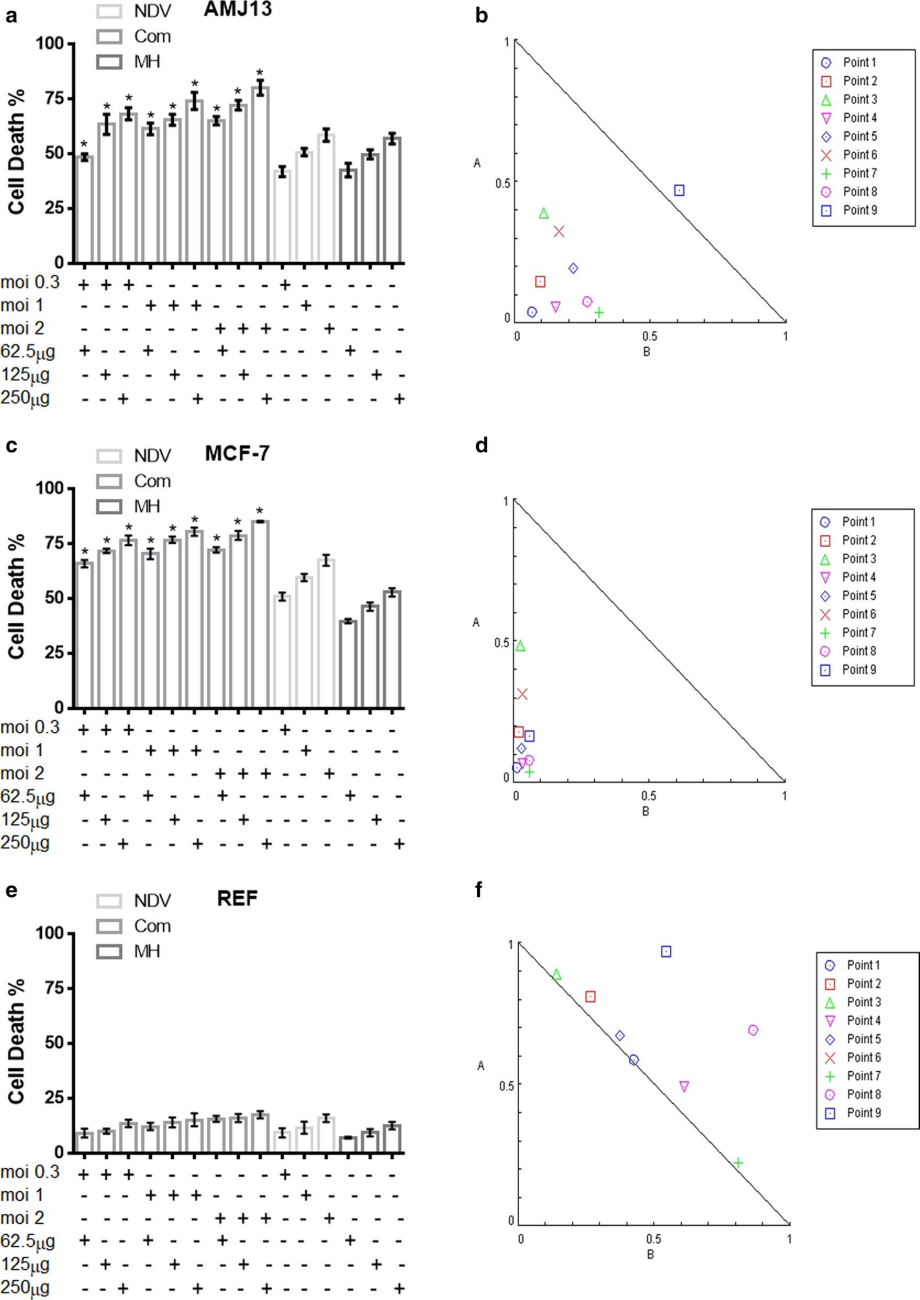
A combination of NDV and MH showed superior anticancer activity in comparison to monotherapies in both AMJ13 and MCF-7 breast cancer cells. However, there was no enhanced toxicity against non-cancerous REF cells. (**a**, **c** and **e**) AMJ13, MCF-7, and REF cells were treated with NDV (0.3, 1, and 2 MOI) and with MH (62.5, 125, and 250 μg/ml), then cell viability was measured by MTT assay. **d**–**f** Illustrations of normalized isobologram of nonconstant combination ratios were measured by the Chou-Talalay method, where CI value quantitatively defines synergism. (CI < 0.9), additive effect (CI = 0.9–1.1) and antagonism (CI > 1.1). All data shown are mean ± SEM (*P < 0.05 compared to mono-treatments) data from three different experiments

In order to investigate the effects of oncolytic NDV and MH combination therapy, we examined the cytotoxicity ratio of the NDV (0.3, 1, and 2 MOI) and for the MH (62.5, 125, and 250 μg/ml). Synergism was observed in all combined doses against both breast cancer (AMJ13 and MCF7) cell lines (Fig. 2a, b). Whereas no synergism relationships were detected among treatments against the non-cancerous REF cell line (Fig. 2e).

The CI was estimated from the dose–effect data of single and combined treatments by using CompuSyn Isobologram. CI < 1 indicates synergism; CI = 1 to 1.1 indicates an additive effect; and CI > 1.1 indicates antagonism. The AMJ13 cell line had CI < 1 in eight combination points, indicating a synergistic effect or interaction between NDV and MH. The additive effect was seen in one combination point (Table 1A) and (Fig. 2a, b). The combination points for the combined treatment to MCF-7 cell line indicated a synergistic effect or interaction between NDV and MH at all points (Table 1B) and (Fig. 2c, d). In the normal REF cell line, all combination points were CI > 1, indicating antagonism and additive effect, which is a neglected effect as there was no killing effect reached 50% at all tested concentrations (Table 1C) and (Fig. 2e, f).

**Table 1:** Cytotoxicity of NDV and MH combination or alone against AMJ13, MCF-7, and REF cells

Points	NDV (MOI)	MH (µg/ml)	CI	Effect
A-AMJ13				
1	2	250	0.10595	Synergism
2	2	125	0.24635	Synergism
3	2	62.5	0.49989	Synergism
4	1	250	0.20755	Synergism
5	1	125	0.41359	Synergism
6	1	62.5	0.49220	Synergism
7	0.3	250	0.35256	Synergism
8	0.3	125	0.34603	Synergism
9	0.3	62.5	1.07844	Additive
B-MCF-7				
1	2	250	0.06841	Synergism
2	2	125	0.20042	Synergism
3	2	62.5	0.51245	Synergism
4	1	250	0.10016	Synergism
5	1	125	0.15299	Synergism
6	1	62.5	0.34948	Synergism
7	0.3	250	0.09957	Synergism
8	0.3	125	0.13597	Synergism
9	0.3	62.5	0.22648	Synergism
C-REF				
1	2	250	1.01291	Additive
2	2	125	1.07549	Additive
3	2	62.5	1.03328	Additive
4	1	250	1.10258	Antagonism
5	1	125	1.04811	Additive
6	1	62.5	1.38593	Antagonism
7	0.3	250	1.03584	Additive
8	0.3	125	1.56003	Antagonism
9	0.3	62.5	1.51414	Antagonism

CI was measured by CompuSyn software. (A) CI values from the treatment of AMJ13 cancer cells; (B) CI values from treatment in MCF-7 cancer cells; (C) CI values from the treatment of non-cancer cells (REF)

### Apoptosis and morphological changes of breast cancer and normal cell lines

**Fig. 3:**
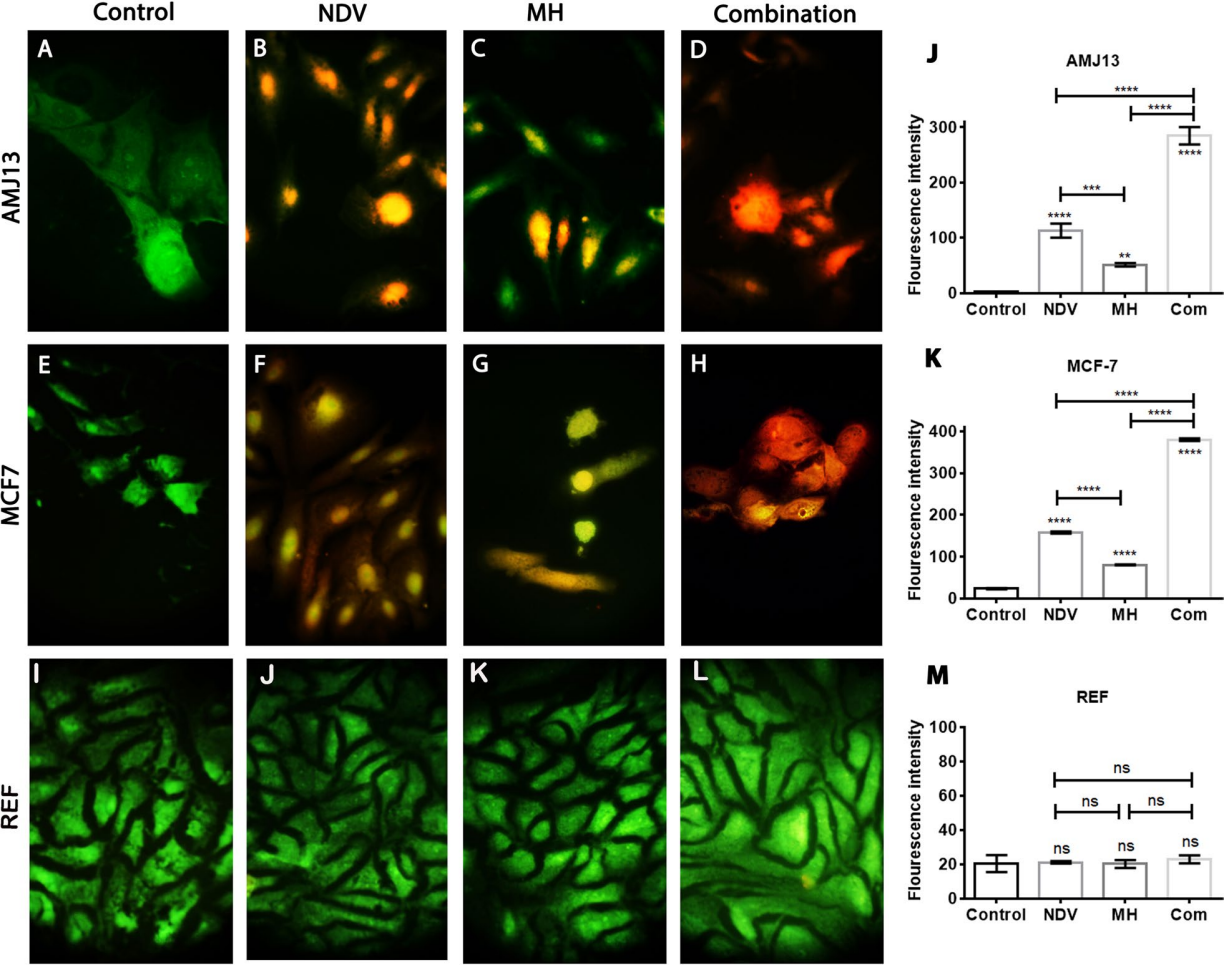
Investigation the MH-NDV combined therapy to induce apoptosis in treated cells using acridine orange and propidium iodide: (**a–d**) AMJ13 cancer cells indicated that MH-NDV induces apoptosis as evidenced red-stained cells, Untreated control cells emitting green fluorescence. **e**–**h** MCF-7 cells were showing that the number of apoptotic cells is higher in the combination treatment. **i**–**l** There was no effect against REF cells by combination therapy using MH-NDV. **j**, **k** MH-NDV treated AMJ13, and MCF-7 breast cancer cells expressed significant apoptotic cell death in comparison to monotherapies and the untreated control cells. **m** no significant changes in all treatment modalities. Values represent the (mean ± SD). *P < 0.05, **P < 0.01, ***P < 0.001 and ****P < 0.0001. The magnification of all images was 400×

The morphological changes exhibited by treated cells after 72 h of treatment were attributed to an intense cytopathic effect for the combination therapy. AO/PI assay, as seen under the fluorescent microscope, showed the untreated apoptotic cells appeared green (viable cells), whereas the apoptotic treated cells with MH and NDV appeared yellow or orange (dead cells) (Fig. 3). Morphological changes and apoptosis were more intense in the cells treated by combined NDV and MH than in those treated with NDV or MH alone. This result indicated that the synergism between the inhibitor and virus enhanced the morphological changes and the percentage of apoptosis in cancer cells (Fig. 3j, k). We noticed weaker or less intense morphological changes and apoptosis in the normal REF cell line (Fig. 3m) than in the breast cancer cell lines treated with MH, NDV, and their combination.

### Hexokinase activity assay analysis indicates strong HK enzyme activity reduction in both breast cancer cell lines treated by MH-NDV combination therapy but not in normal embryonic cells

**Fig. 4:**
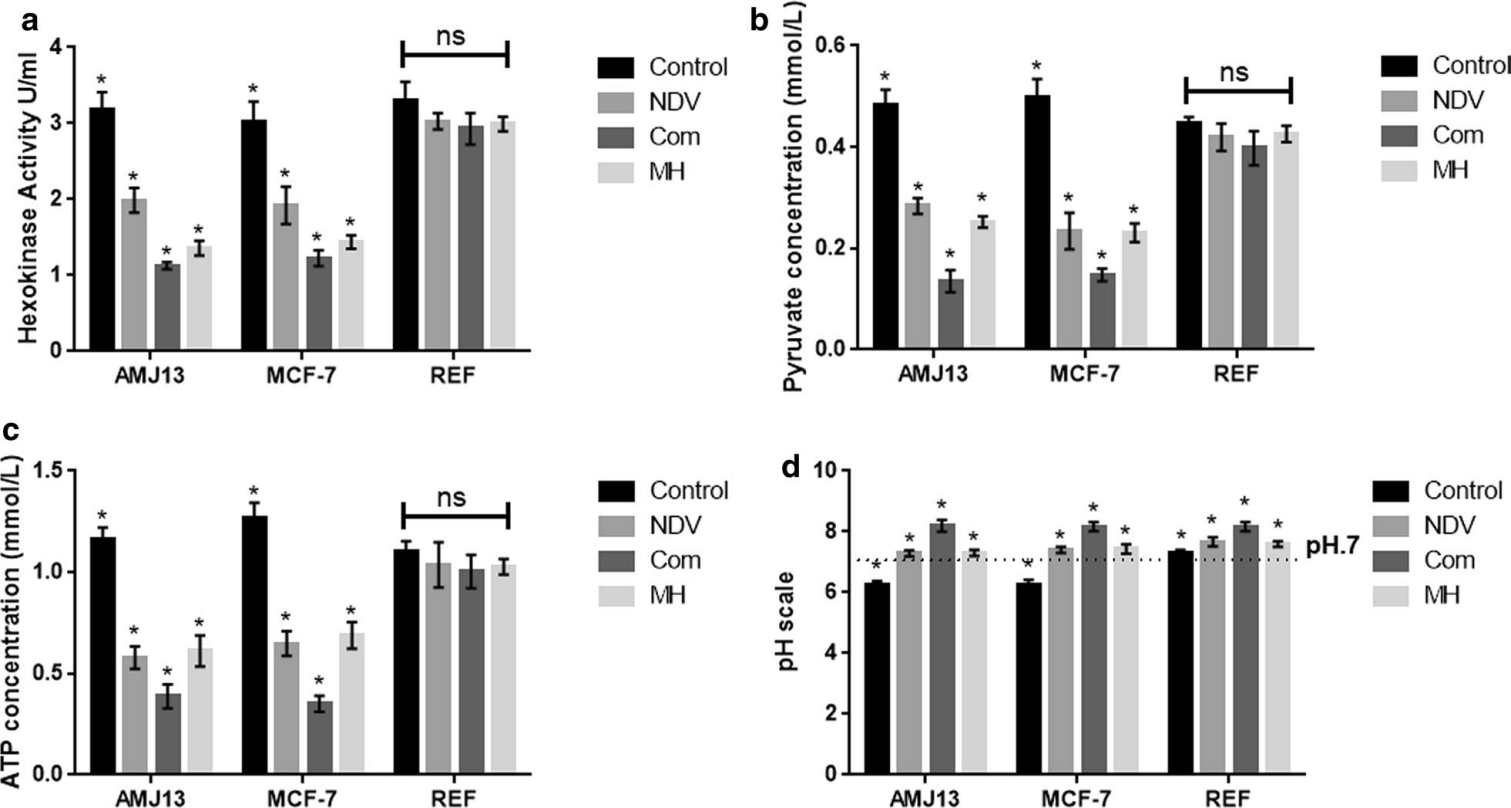
MH–NDV combination efficiently inhibits glycolysis products in the treated breast cancer cells but not in normal cells in comparison to monotherapies. **a** MH–NDV combined therapy induces a significant decrease in the activity of hexokinase in AMJ13 and MCF-7 cancer cell lines. At the same time, there is no significant reduction in normal REF cells (**b**, **c**) measurement of pyruvate and ATP levels in cancer cells significantly reduced in MH-NDV combination therapy in comparison to both monotherapies and untreated control cells. However, the reduction in the normal REF cells was not significant. **d** Measurements of pH levels in AMJ13 and MCF-7 breast cancer cells indicate that MH-NDV treated cells supernatant was more alkaline in comparison to both monotherapies significantly, while untreated control cells showing acidity as well as the control and treated non-cancerous REF cells. All data shown are mean ± SEM (*P < 0.05) data from three different experiments using unpaired *t*-test, stars on MH and NDV column means it is significant in compare to each other and star on the com means it is significant in compare to monotherapies (MH and NDV). Star on the control column means it is significant when compared to MH, NDV and Com

In the current experiment, we quantified and analyzed the HK enzyme activity in both breast cancer cell lines and the normal embryonic cells. The HK enzyme was evaluated for the comparison of treated and untreated cells at 72 h (Fig. 4a). We identified a significant reduction in the HK enzyme activity in all the treatment modalities in the cancer cells but not in the normal cells. The results showed that the HK enzyme activity was significantly reduced in the combination of MH-NDV treated breast cancer cells in comparison to the single treatment modalities.

### MH–NDV combination efficiently inhibits glycolysis products in the treated breast cancer cells but not in normal cells

Further investigation of the mechanism by which MH–NDV inhibits breast cancer cell proliferation was done through examination whether MH–NDV efficiently decreases cancer cell glycolysis products pyruvate, ATP, and acidity (represent lactic acid) compared to monotherapies. Remarkably, MH–NDV induces a significant reduction on the levels of pyruvate, ATP, and acidity in both breast cancer cell lines but not in normal embryonic REF cells (Fig. 4b–d).

## Discussion

Malignant cells use glycolysis as their main source of energy. The in vitro results of this study showed that by increasing the concentration of MH and the MOI of NDV, there is an increase in cytotoxicity and enhanced antiproliferation effect against breast cancer cell lines but not in normal cells. MH-NDV combination, in turn, found to reduce HK activity, pyruvate, ATP, and acidity levels. Our findings are similar to previous results showing that NDV in combination with 2-Deoxyglucos, may inhibit glycolysis as the main pathway to induce breast cancer-killing effect [30]. Moreover, inhibiting glycolysis with 3-bromopyruvate and 2-deoxyglucose results in mitochondrial pathway-induced apoptosis in cancer cells [37]. We found that MH and NDV had a significant effect on breast cancer cells but a non-significant effect on normal cells. The combination of NDV and MH had the strongest effect on cancer cell lines in comparison to monotherapies. CI values revealed high synergism between MH and NDV (CI < 1) in both MCF-7 and AMJ13 cell lines. All values for the REF cell line showed no synergism and they have neglected cytotoxicity due to the fact of absence any death percentage above 50%.

AO/PI assay observations revealed that combination therapy was the best inducer for apoptosis, and that was compatible with our previous results. NDV induces apoptosis in virus-treated cells [38]. Furthermore, it’s found that glycolysis inhibitors enhance apoptosis and mitochondrial damage [38]. Apoptosis is essential for the prevention of tumor formation and cancer growth [39]. Our results support the aim of our study.

In addition, our results demonstrated that MH-NDV inhibits the glycolytic pathway in MCF-7 and AMJ13 cell lines by suppressing the activity of HK more than the specific inhibitor alone and more than NDV treatment alone. Wang et al. [40] described HK2- targeting modulate Warburg effect to stimulate cancer cell apoptosis. HK inhibition can cause ATP depletion, thus resulting in insufficient energy supply for cancer cell mitosis, proliferation, and invasion, as previously described [39]. HK activity in breast cancer cell lines was lower than that in control cells, and a non-significant reduction in HK activity was noticed in the REF normal cell line.

Patra et al. [14] discovered that the deletion of the HK2 gene in adult mice does not considerably disturb normal tissues. Moreover, MH is a specific inhibitor for HK II [19], and normal cells rely more on HK I [41] this explains why REF cells showed a mild non-significant reduction in HK activity. There are several studies described the role of HK2 as essential in tumor initiation and development in breast cancer; therefore, HK2 deletion as a cancer treatment have therapeutic value with no adverse physiological side effects [14, 42]. This effect prevented cancer cell proliferation. Thus, the targeting of this key enzyme by inhibitors inhibits glycolysis and therefore suppresses cancer cell proliferation [43]. We noticed a significant reduction in pyruvate concentration, after treatment with the combination in breast cancer cell lines, and a non-significant relation in the REF normal cell line after 72 h of treatment. Given that pyruvate concentration depends on the action of HK in the glycolysis pathway [44], the decrease in HK resulted in a deficiency in pyruvate concentration, which confirmed by our experiment.

In correlation with previous experiment results, we noticed that the MH-NDV combination suppresses the generation of intercellular ATP. We found that the ATP concentrations in AMJ13 and MCF-7 cell lines treated with MH and NDV were significantly lower than those of the control. Reducing ATP levels effectively inhibits energy metabolism in MCF-7 and MDA-MB-231 cells. When glycolysis is inhibited, lactate production is completely terminated, and intracellular ATP concentration abruptly decreases [45]. These phenomena are consistent with the results of our study. Our results also provided further evidence that glycolysis is increased in transformed cells (cancer cells) in comparison with that in normal cells, which reported before [46].

The pH values of treated breast cancer cell lines secretions were higher than those of the control. A non-significant effect was detected in the normal REF cell line after treatment with MH and NDV. Acidity decreased because lactate concentration decreased as a result of reduced pyruvate concentration and inhibited the growth and proliferation of cancer cells. It is proved that lactate deficiency reduces the acidity of the cell environment [38]. This effect is favorable for preventing cancer cell proliferation because tumors or cancer cells grow in acidic environments.

## Conclusion

Our results revealed that MH, NDV, and their combination could inhibit the growth and proliferation of breast cancer cell lines by increasing cytotoxicity through inhibiting the glycolysis pathway and inducing apoptosis. These effects were attributed to specific reductions in HK activity by the MH-NDV combinations leading to a decrease in pyruvate, ATP, and micro-environmental acidity. In addition, our results showed that the combined MH and NDV treatment had potent cytotoxic effects against breast cancer cell lines but not against the normal REF cell line that proved safety. This treatment affected and enhanced cancer cell apoptosis as a result of glycolysis inhibition. Our investigation proved the effect of MH and NDV and the strong effect of synergism between these compounds as the best therapy for cancerous cells in vitro. This strategy has potential applications as an effective cancer treatment. Our observations will provide new insight into the development of therapeutic strategies for breast cancer and other types of cancer in the near future.

## Data Availability

Data available on request from the authors, the data that support the findings of this study are available from the corresponding author upon reasonable request.
